# TGF-β/SMAD signaling regulation of mesenchymal stem cells in adipocyte commitment

**DOI:** 10.1186/s13287-020-1552-y

**Published:** 2020-01-29

**Authors:** Sheng-Nan Li, Jia-Fa Wu

**Affiliations:** 1 School of Medicine, Henan Polytechnic University, Jiaozuo, 454000 Henan China; 2 School of Food and Bioengineering, Henan University of Science and Technology, Luoyang, Henan China

**Keywords:** TGF-β/SMAD signaling, Commitment, Mesenchymal stem cells (MSCs)

## Abstract

Adipocytes arising from mesenchymal stem cells (MSCs) requires MSC adipocyte commitment and differentiation of preadipocytes to mature adipocytes. Several signaling and some cytokines affect the adipogenesis of MSCs. This review focuses on the roles of TGF-β/SMAD signaling in adipocyte commitment of MSCs. BMP4 and BMP7 signaling are sufficient to induce adipocyte lineage determination of MSCs. The roles of BMP2, TGF-β, and myostatin signaling in this process are unclear. Other TGF-β/SMAD signaling such as BMP3 and BMP6 signaling have almost no effect on commitment because of limited research available, while GDF11 signaling inhibits adipocyte commitment in human MSCs. In this review, we summarize the available information on TGF-β/SMAD signaling regulation of MSCs in adipocyte commitment. Deeper study of this commitment mechanism will offer new approaches in treating obesity, diabetes mellitus, and obesity-related metabolism syndrome.

## Introduction

Mesenchymal stem cells (MSCs) are a group of cells with multilineage differentiation potential and self-renewal capacity, which were discovered and isolated from the bone marrow in the 1960s [1, 2]. Many studies have shown that MSCs can be isolated from almost all tissue types such as adipose tissue and muscle tissue because of the ease of their isolation and their extensive expansion rate [3, 4]. MSCs can differentiate into various specialized cell types such as osteoblasts, chondrocytes, myoblasts, and adipocytes under the stimuli of different conditions [5, 6]. Adipogenesis of MSCs is presently the research hotspot in the stem cell field. In recent years, some studies have shown that several signaling and some cytokines affect the adipogenesis of MSCs. Adipocytes arise from MSCs by two steps: MSCs commit to preadipocytes and preadipocytes differentiate into adipocytes. The first step adipocyte commitment is a stage that can be markedly changed, with hypomethylation of unknown specific genes [7–9]. The formation of preadipocytes is an intermediate state between MSCs and adipocytes. While the adipocytes clearly contain fat droplets, the preadipocytes have none. 3T3-L1 cells and 3T3-F442A cells are some of the most common preadipocytes in use at present because of their high adipogenic potential. The second step adipocyte terminal differentiation is that preadipocytes proliferate by mitotic clonal expansion following the input of several hormones, resulting in terminal adipocyte differentiation. At present, the molecular mechanism of the preadipocytes differentiation into adipocytes is clear but little is known about the mechanism involved in the commitment process from MSCs to preadipocytes. Recent studies indicated that the TGF-β superfamily has an important role in regulating the adipocyte commitment of MSCs.

**Fig. 1:**
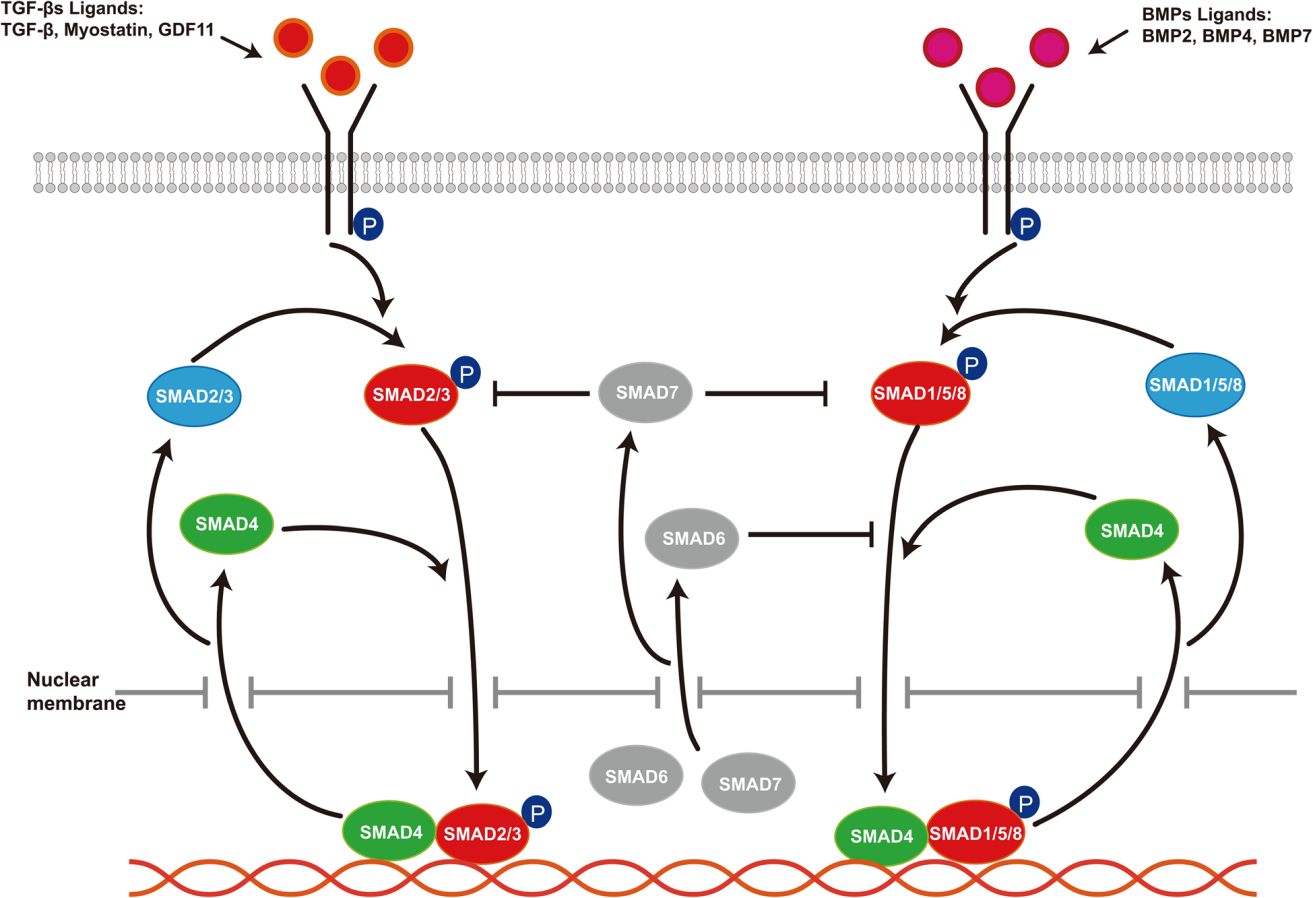
TGF-β/SMAD signaling. TGF-βs ligands such as TGF-β, Myostatin, and GDF11 in TGF-β/SMAD signaling bind to cell membrane receptors to phosphorylate the intracellular downstream SMAD2/3 (R-SMADs), and BMPs ligands such as BMP2, BMP4, and BMP7 phosphorylate the SMAD1/5/8 (R-SMADs). Activated R-SMADs form a complex with SMAD4 and then translocates to the nucleus to regulate the expression of specific genes. After the genes respond to the TGF-β/SMAD signaling, the R-SMADs–SMAD4 complex in the nucleus is depolymerized and they re-enter the cytoplasm. I-SMADs comprise SMAD6 and SMAD7, which negatively regulate TGF-β/SMAD signaling. In the resting state, I-SMADs mainly tend to be in the nucleus. Transcriptionally activated by TGF-β/SMAD signaling, SMAD7 shuttling from the nucleus to the cytoplasm prevents R-SMAD phosphorylation. SMAD6 competes with SMAD1 for binding to SMAD4

TGF-β superfamily signaling regulates the progress of many cellular processes such as apoptosis, inflammation, fibrosis, and adipocyte differentiation [10]. There are 33 human TGF-β superfamily ligands including activin, inhibin, bone morphogenetic proteins (BMPs), growth differentiation factors (GDFs), Lefty, transforming growth factor β (TGF-β), and Nodal [11, 12]. Structural and functional considerations separate the ligands into two subfamilies: TGF-βs and BMPs [12]. TGF-β, activin, myostatin, GDF11,  and Nodal belong to the TGF-βs subfamily, while BMP2, BMP4, and BMP7 belong to the BMPs subfamily. Two pairs of transmembrane serine/threonine protein kinases constitute the TGF-β superfamily receptors: the type I receptors and the type II receptors. Ligand binding to the receptors activates SMAD-dependent pathways (TGF-β/SMAD signaling) or SMAD-independent pathways which mainly promote ERK, JNK, and p38 MAPK kinase signaling [13]. In this review, we focus on the roles of TGF-β/SMAD signaling (Fig. 1) in regulating the adipocyte commitment of MSCs to provide a reference point for future research.

## TGF-β/SMAD signaling

Activin, inhibin, BMPs, GDFs, TGF-β, Nodal, and other ligands involved in TGF-β/SMAD signaling bind to cell membrane receptors to activate the intracellular downstream SMAD family proteins. The SMAD family proteins constitute three functional classes: the receptor-regulated SMADs (R-SMADs), the common partner SMAD (Co-SMAD), and the inhibitory SMADs (I-SMADs) [14, 15]. The R-SMADs comprise SMAD1, SMAD2, SMAD3, SMAD5, and SMAD8. The Co-SMAD only refers to SMAD4. The I-SMADs consist of SMAD6 and SMAD7.

In the resting state, R-SMADs mainly exist in the cytoplasm, Co-SMAD predominantly localizes to both the cytoplasm and nucleus, and I-SMADs mainly tend to be in the nucleus. Upon TGF-βs ligand binding, the receptors recruit and phosphorylate SMAD2/3, and SMAD1/5/8 by BMPs ligands. After C-terminal phosphorylation of the R-SMADs, these receptor-activated R-SMADs (SMAD2/3 or SMAD1/5/8) are released from the receptor complex to form a trimeric complex consisting of two R-SMADs and a SMAD4 molecule, which then translocates to the nucleus to regulate the expression of specific genes [13, 16, 17]. After these genes respond to the TGF-β/SMAD signaling, the R-SMAD–SMAD4 complexes in the nucleus are dephosphorylated by dephosphorylase, and then are depolymerized and re-enter the cytoplasm through the nuclear pore, realizing the recycling of the R-SMADs and SMAD4 [18]. I-SMADs negatively regulate TGF-β/SMAD signaling. When the ligands bind to the receptors, SMAD7 shuttles from the nucleus to the cytoplasm, and binding to the type I receptor prevents R-SMAD phosphorylation to inhibit specific gene expression [19]. SMAD6 acts in a different manner to SMAD7. After the receptors are activated, SMAD6 undergoes shuttling from the nucleus to the cytoplasm and competes with SMAD1 for binding to SMAD4, serving as an inhibitor of TGF-β/SMAD signaling [20].

## TGF-β/SMAD signaling regulation of MSCs in adipocyte commitment

**Fig. 2:**
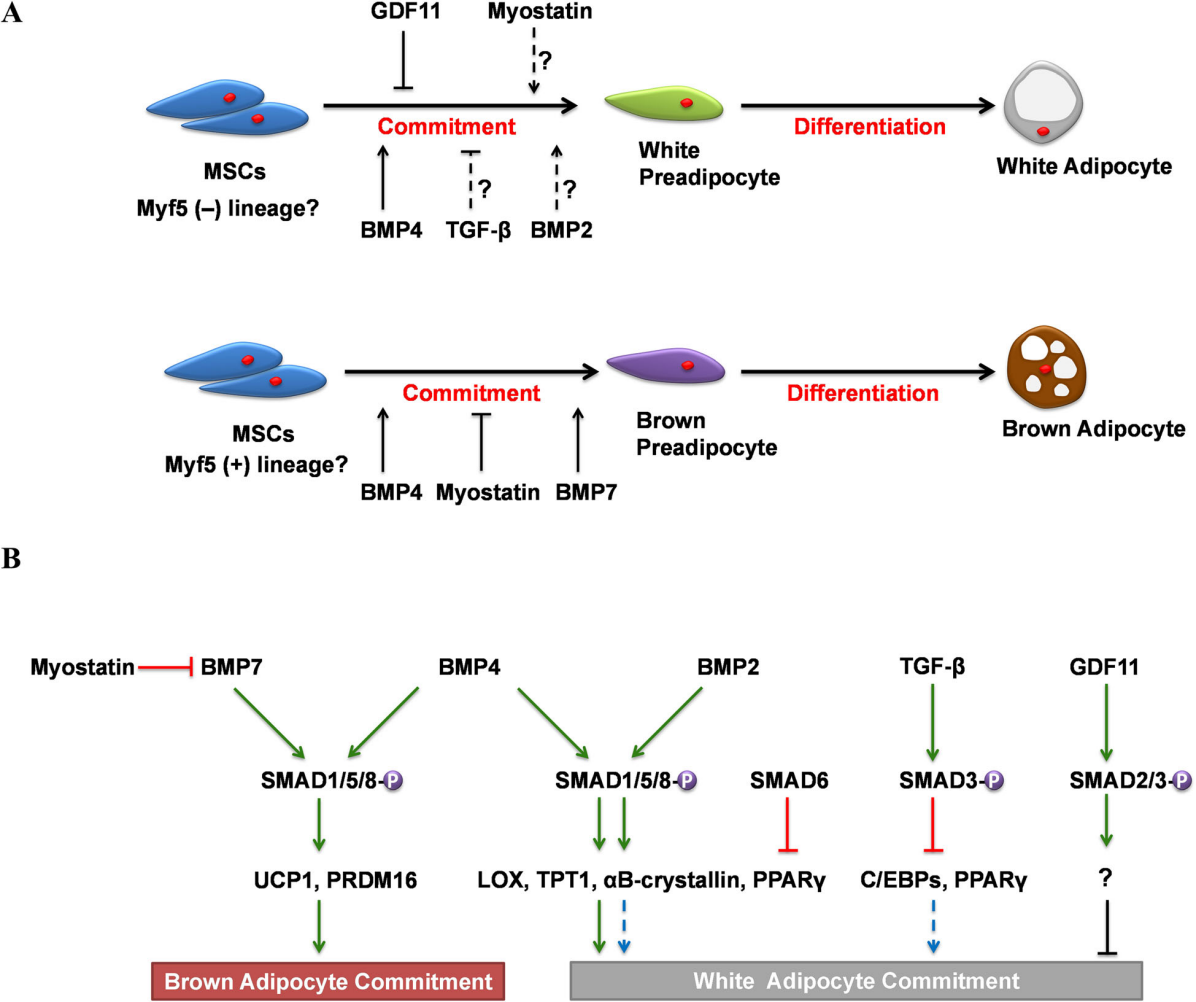
Schematic summary of TGF-β/SMAD signaling action on the adipocyte commitment of MSCs. **a** Adipocytes arise from MSCs by two steps: MSCs commit to preadipocytes and these preadipocytes differentiate into adipocytes. The adipocyte origin is more complex. The Myf5^−^ lineage mainly gives rise to white preadipocytes, and the Myf5^+^ lineage gives rise to brown preadipocytes, but may not be absolute. BMP4 and BMP7 signaling are sufficient to induce adipocyte lineage determination. GDF11 inhibits the commitment of human MSCs. The roles of BMP2, TGF-β, and myostatin signaling in this process are unclear. It is uncertain whether they promote adipocyte commitment or inhibit it. **b** The major regulating mechanism of TGF-β/SMAD signaling in adipocyte commitment in MSCs

Adipose tissue dysfunction can lead to obesity, fatty liver, dyslipidemia, and type II diabetes [8, 21, 22]. According to its color, function, and anatomy, adipose tissue is sub-classified as white adipose tissue (WAT), brown adipose tissue (BAT), and beige adipose tissue. WAT in humans is located abdominally and subcutaneously for lipid storage. BAT, which expresses uncoupling protein 1 (UCP1), is mostly located in the neck and supraclavicular regions of adult humans for thermogenesis [23]. When the body is under the stimulation of adrenalin or cold exposure for a long time, some cells in the white adipose tissue will express high levels of UCP1, stimulate thermogenesis, and undergo “browning” to adapt to the environmental change. These cells are called beige adipose cells [24, 25]. A lot of beige adipose cells compose the beige adipose tissue. Detailed molecular comparisons of white, brown, and beige adipose tissue have identified several specific markers (Table 1) [26, 27]. Adipose tissue is composed of plentiful adipocytes, which are derived from MSCs (MSCs involved in the study are listed in Table 2), cells that have multilineage differentiation capacity and reside in the vascular stroma of adipose tissue as well as in the bone marrow [50]. The Myf5^−^ lineage mainly gives rise to white preadipocytes, and the Myf5^+^ lineage gives rise to brown preadipocytes, but may not be absolute. Some studies confirmed that brown adipocytes and a subset of white adipocytes originated from Myf5^+^ lineage [51]. Most studies favor the brite adipocytes originate from white adipocytes, but some studies suggest brite adipocytes may originate from mesenchymal progenitor cells [52–55]. The formation of mature adipocytes requires adipocyte commitment of MSCs and adipocyte differentiation. It is now established that TGF-β/SMAD signaling has a dual role in the adipocyte differentiation process. BMPs signaling promotes the mature of adipocytes while TGF-βs signaling inhibits it. In recent years, several studies have shown that TGF-β/SMAD signaling has a critical role in regulating the adipocyte commitment of MSCs (Fig. 2).

**Table 1:** The comparisons of white, brown, and beige adipose tissue

Types of adipose tissue	Specific gene	Anatomy	Lipid droplet	Specific markers	Function
White adipose tissue	*UCP1* negative	Abdomen subcutis	A large unilocular lipid droplet	*Tcf21*	Lipid storage, secrete hormones and cytokines
Brown adipose tissue	*UCP1* positive	Neck supraclavicular regions	Multilocular lipid droplets	*Zic1*	Thermogenesis secrete hormones and cytokines
Beige adipose tissue	*UCP1* positive	WAT	Multilocular lipid droplets	*Tbx1*, *Epsti1 Cd137*, *Tmem26*	Thermogenesis

*WAT*, white adipose tissue; *UCP1*, uncoupling protein 1; *Tcf21*, transcription factor 21; *Zic1*, zinc finger protein of the cerebellum 1; *Tbx1*, T-box 1; *Epsti1*, epithelial stromal interaction 1; *Cd137*, tumor necrosis factor receptor superfamily, member 9; *Tmem26*, transmembrane protein 26

**Table 2:** Cell type involved in the regulation of adipocyte commitment under TGF-β/SMAD signaling

Cell type (MSC)	Species	Signaling	References
C3H10T1/2	Mouse	BMP4 signaling	[8, 28–32]
		BMP2 signaling	[28–31, 33, 34]
		BMP7 signaling	[32, 35]
		Myostatin signaling	[36–38]
Human adipose stem cell (hASC)	Human	BMP4 signaling	[32, 39–41]
		BMP2 signaling	[42]
		BMP7 signaling	[41, 43]
Mouse bone marrow stromal stem cell (mouse BMSC)	Mouse	BMP7 signaling	[44]
		TGF-β signaling	[45]
Human bone marrow stromal stem cell (human BMSC)	Human	BMP7 signaling	[46]
		TGF-β signaling	[47, 48]
		Myostatin signaling	[49]

*MSC*, mesenchymal stem cell; *cell type (MSC)*, cell type involved in the study; *species*, species involved in study; *signaling*, TGF-β/SMAD signaling involved in the regulation of adipocyte commitment; *references*, references related to the study in this table

### BMP4 signaling in the adipocyte commitment of MSCs

The role of BMP4 signaling has been validated in the commitment process of MSCs. C3H10T1/2 cells were isolated from C3H mice in 1973 and can differentiate into various cell types such as adipocytes, osteocytes, chondrocytes, and myoblasts [6, 56]. C3H10T1/2 cells can be faithfully considered as MSCs in terms of their multidirectional differentiation and strong proliferative ability. Their easy isolation, long lifetime in culture, and low culture cost, as well as the absence of ethical concerns, have resulted in the widespread use of these cells. Several studies indicate that BMP4 can induce pluripotent C3H10T1/2 cells to commit to the adipocyte lineage [28, 29]. Upon BMP4 ligand binding to the receptors BMPr1A and BMPr2, the receptors phosphorylated SMAD1/5/8, which formed a complex with SMAD4. The complex translocated to the nucleus to regulate *LOX* (lysyl oxidase), *TPT1* (translationally controlled tumor protein 1), and *αB-crystallin* which are target genes of BMP4 signaling in the adipocyte commitment of C3H10T1/2 cells [30, 31]. Treatment of C3H10T1/2 cells with BMP4 revealed their capacity to differentiate into adipocytes, and the expression of adipocyte markers CCAAT/enhancer binding protein-α (C/EBPα), peroxisome proliferator-activated receptor-γ (PPARγ), and adipocyte protein 2 (AP2) were detected. C3H10T1/2 cells pretreated with BMP4 were subcutaneously implanted into athymic mice, and the cells developed into tissue that was undistinguishable from adipose tissue [28]. The research of Bowers et al. also confirmed that BMP4 signaling was sufficient and necessary to induce adipocyte lineage determination in C3H10T1/2 cells [8]. A33 cells are a committed preadipocyte cell line derived from C3H10T1/2 cells after 5-azacytidine treatment [8]. Exposure of A33 cells with noggin, a BMP4 binding antagonist, blocked the subsequent differentiation, indicating BMP4 is necessary to maintain commitment [8]. BMP4 also plays a crucial role in regulating human adipose tissue stromal cell commitment. Human adipose tissue stromal cells are also referred to as human adipose stem cells (hASCs), which are a subset of stromal vascular fraction (SVF) cells of adipose tissue [57]. The hASCs are multipotent cells that can differentiate into several different lineages of cells, such as adipocytes, chondrocytes, and osteoblasts. Because hASCs exhibit properties similar to MSCs, some researchers advise that the two cell lines are identical [58]. Inhibition of BMP4 signaling decreased adipogenesis in hASCs [39]. SMAD1/5/8 is phosphorylated by activated BMP receptors. It then associates with SMAD4 and the complex translocates to the nucleus where it regulates gene expression. Overexpression of constitutively active BMP receptors induced adipocyte commitment without exogenous BMP4, whereas overexpression of a dominant-negative BMP receptor suppressed the commitment that was induced by exogenous BMP4 [30]. Also, knockdown of *SMAD4* impeded adipocyte commitment by exogenous BMP4. Treatment with 40 ng/mL BMP4 triggered the adipocyte commitment of hASCs. The committed cells differentiation into adipocytes was associated with increased activation of PPARγ, APM1, AP2, and GLUT4 [40]. BMP4 has been shown to promote the commitment of the early precursor cells. WISP2, as a secreted protein of adipose precursor cells, can form a complex with ZFP423 without BMP4 stimulation. The addition of BMP4 activated the phosphorylation of SMAD1/5/8, dissociated the WISP2/ZFP423 complex, allowed ZFP423 to enter the nucleus for PPARγ activation, and committed the cells to the adipose lineage [59]. To date, there have been few studies of BMP4 in BAT. Some research had found that BMP4 combined with BMP7 induced expression of the terminal BAT-specific marker UCP1 in hASCs [32]. BMP4 also induced the formation of brown adipocytes in C3H10T1/2 cells [32]. The expression of UCP1 and the early regulator of brown fat fate protein PRDM16 were induced when C3H10T1/2 cells were pretreated with 20 ng/ml recombinant human BMP4. Implantation of C3H10T1/2 cells pretreated with BMP4 into nude mice resulted in the development of UCP1-positive brown adipocytes. BMP4 increased the white-to-brown transition and the expression of UCP1 and decreased the expression of the white specific marker transcription factor 21 (TCF21) in hASCs [41]. It also enhanced insulin sensitivity, adipocyte cell number, and whole-body energy expenditure by browning subcutaneous adipose tissue in mature mice [60, 61].

### BMP2 signaling in the adipocyte commitment of MSCs

BMP2 signaling  in the adipocyte commitment of MSCs remains unclear. Several studies found that BMP2 signaling can induce pluripotent C3H10T1/2 cells to commit to the adipocyte lineage [29–31, 33]. There was a certain degree of plasticity between adipogenesis, chondrogenesis, and osteogenesis, and low-level addition of BMP2 to C3H10T1/2 cells favored adipogenesis [29, 33]. BMP2, just like BMP4, activated the expression and phosphorylation of SMAD1/5/8, which formed a complex with SMAD4. The complex translocated to the nucleus to regulate *LOX*, *TPT1*, and *αB-crystallin* in the adipocyte commitment of C3H10T1/2 cells [30, 31]. Knockdown of *SMAD4* expression prevented the adipocyte commitment of C3H10T1/2 cells by BMP2 [30]. BMP2 induced the adipocyte commitment of C3H10T1/2 cells by induction of PPARγ expression through activation of SMAD1 and p38 kinase, suggesting a central role of PPARγ in this commitment event [34]. SMAD6 blocked PPARγ expression and adipocyte commitment of C3H10T1/2 cells induced by BMP2. BMP2 (100 ng/ml) regulated SMAD signaling, decreased proliferation, and exhibited a donor-dependent dual role, inducing osteogenic or adipogenic differentiation in hASCs [42]. The expression of alkaline phosphatase, an osteoblast-specific marker, as well as the mineralization levels were notably enhanced in some donor cell lines by BMP2 stimulation, while diminishing lipid formation in other cell lines. The hASCs derived from older donors had higher potential to demonstrate BMP2-induced adipogenesis than cells from younger individuals. BMP2 induced SMAD1/5 phosphorylation, activation, and translocation to the nucleus to regulate adipocyte commitment, and the expression of adipogenic marker AP2 was remarkably detected [42]. The relevance of the origin of BMP2 production is that it influences the adipocyte commitment of hASCs. BMP2 produced in mammalian cells, Chinese hamster ovary (CHO) cells, induced higher levels of lipid formation compared with BMP2 of *Escherichia coli* origin [42]. Conversely, some studies have shown that BMP2 inhibits adipocyte commitment [28, 62, 63]. Tang et al. found that adding 50 ng/ml BMP2 to C3H10T1/2 cells until post-confluency followed by standard adipocyte differentiation failed to cause an accumulation of triglyceride or the expression of the adipocyte-specific marker AP2 [28]. Thus, it can be concluded that BMP2 inhibits adipocyte commitment in C3H10T1/2 cells. However, the resulting research of Tang et al. considered that BMP2 can induce pluripotent C3H10T1/2 cells to commit to the adipocyte lineage, as shown previously [30, 31]. Despite some common ground, we identified some disagreements through a comparative study of these papers and analyses. C3H10T1/2 cells in these studies were all plated at low density with purified recombinant BMP2 at the same concentration of 50 ng/ml. C3H10T1/2 cells were induced to differentiate using the adipocyte differentiation protocol after the cells reached post-confluency. The difference was that they added different concentrations of an adipogenic induction cocktail consisting of insulin, dexamethasone, and 3-isobutyl-1-methylxanthine. The later research of Tang et al. changed DMEM containing 10% calf serum (CS) for DMEM containing 10% fetal bovine serum (FBS) in the process of differentiation because FBS may have some effects on adipocyte commitment. Future research should focus on the effect of the different components between CS and FBS in adipocyte commitment. Other studies have shown that BMP2 enhanced osteoblast commitment in a bone marrow stromal cell line by increasing the expression of osteoblast-specific markers OSF2/CBFA1 and mineralized nodule formation, concomitantly inhibiting adipocyte maturation [62, 63]. The effects of BMP2 on adipocyte differentiation reduced adipogenesis and decreased the levels of leptin and the formation of cytoplasmic lipid droplets. It should be noted that in the article, the multipotent murine bone marrow stromal cell line BMS2 cannot be regarded as MSCs, because up to 40% or more of the BMS2 population could spontaneously differentiate into adipocytes only in the presence of adipogenic induction cocktail. In conclusion, BMP2 may guide the commitment destiny of MSCs depending on the donor line, the concentrations of an adipogenic induction cocktail, and the type of serum added to DMEM. Further studies are required to clarify how BMP2 regulates the commitment of MSCs.

### BMP7 signaling in the adipocyte commitment of MSCs

Many studies have demonstrated that BMP7 has an important role in brown adipocyte lineage determination [32, 35, 36, 41, 44, 46]. BMP7 triggered the commitment of C3H10T1/2 cells to a brown adipocyte lineage with a significant increase in lipid accumulation and expression of UCP1 [32, 35]. BMP7 stimulated cell proliferation and differentiation in mouse bone marrow stromal stem cells (BMSCs) and human adult MSCs [44, 46]. Low concentrations stimulated adipocyte differentiation, whereas it was inhibited at higher concentrations under BMP7 treatment in mouse BMSCs [44]. BMP7 promoted the adipogenic differentiation of human BMSCs but not the osteogenic or chondrogenic lineage development in a high-density micro-mass culture [46]. BMP7 or BMP4 also induced adipogenic differentiation by lipid accumulation and PPARγ expression, while inducing the white-to-brown transition by increasing UCP1 expression and decreasing the expression of white specific marker TCF21 in hASCs dependent on the donors [41]. Implantation of BMP7-treated C3H10T1/2 cells subcutaneously into the sternal region of athymic nude mice resulted in the development of adipose tissue containing brown adipocytes in vivo [35]. Although *BMP7* knockout mice died shortly after birth, BAT mass was reduced and there was an almost complete absence of UCP1 in the knockout newborns [35]. Mice injected with adenovirus expressing BMP7 showed a significant increase in brown fat mass and energy expenditure [35]. BMP7 also drove human adipogenic stem cells to form metabolically active beige adipocytes [43]. Human adipogenic stem cells are also referred to hASCs. Exposure of hASCs to human BMP7 was associated with a significant increase in UCP1 expression and beige specific marker gene expression as well as metabolic activity [43].

### TGF-β signaling in the adipocyte commitment of MSCs

TGF-β is another important molecule that regulates the adipocyte commitment of MSCs. TGF-β signaling inhibited adipocyte commitment via SMAD3 signaling [64]. TGF-β inhibited C/EBPs and PPARγ expression and induced PPARγ phosphorylation, resulting in inhibition of the adipocyte commitment of BMSCs [45]. Deleting TGF-β in mesenchymal cells resulted in a marked expansion of adipocytes in the bone marrow of mice and significantly increased the expression of PPARγ and FABP4 associated with adipocyte commitment, while osteoblasts were significantly reduced [65]. Since PPARγ is a master regulator in adipocyte commitment, TGF-β suppresses the adipocyte commitment of MSCs through phosphorylation of PPARγ. Several articles focused on the modulation of TGF-β signaling in adipogenic differentiation of hASCs and the multipotent mesenchymal stromal cells misleading the MSC origin. In these studies, TGF-β inhibited adipogenic differentiation in these cells [66, 67]. However, it should be noted that the hASCs and the multipotent mesenchymal stromal cells in these articles cannot be regarded as MSCs because more of the population could spontaneously differentiate to adipocytes with exposure to only adipogenic induction cocktail. In a sense, the cells can be regarded as preadipocytes. We should not only focus on the surface but also think carefully about what MSCs are, perhaps according to their property. Although some studies have reported that TGF-β suppresses the adipocyte commitment of MSCs, recent studies showed that TGF-β promotes adipocyte commitment. A single pulse dose treatment with TGF-β during the commitment phase led to increased adipogenesis of human BMSCs, while continuous treatment during the whole phase (commitment phase and differentiation phase) inhibited adipogenesis [47]. The reason could be TGF-β promoted the first step adipocyte commitment, but inhibited the second step adipogenic differentiation of BMSCs by repressing the function of C/EBPβ and C/EBPδ during the differentiation phase [68]. TGF-β promoted osteogenic and adipogenic differentiation of a clone isolated from human  BMSC lines, which have limited differentiation capacity, suggesting an epigenetic change rather than a gene mutation [48].

### Myostatin signaling in the adipocyte commitment of MSCs

Myostatin, also called GDF8, is a well-known regulator of myogenesis inhibition. Some researchers found that myostatin can promote adipogenesis in MSCs. C3H10T1/2 cells were incubated in regular medium with graded concentrations of myostatin and found that there were many oil droplets present after a 2-week incubation, as visualized by Oil Red O staining [36]. Myostatin failed to trigger adipogenesis in the 3T3-L1 preadipocyte cell line, but it was also a substitute for dexamethasone, which can trigger adipogenesis and induce small and apparently immature adipocytes of C3H10T1/2 cells [37]. Myostatin gene (*Mstn*) knockout in mice (*Mstn*^−/−^ mice) increased myogenesis and decreased adipogenesis [69, 70]. *Mstn*^−/−^ mice had reduced adipogenesis and consequently decreased leptin secretion and C/EBPα and PPARγ levels [69]. *Mstn*^−/−^ mice also had suppressed obesity and glucose metabolism than control mice [70]. The molecular mechanism of the phenomenon by which *Mstn*^−/−^ mice had reduced adipogenesis was that the absence of *Mstn* resulted in enhanced peripheral tissue fatty acid oxidation and brown adipose formation in white adipose tissue [71]. Some studies found that myostatin inhibited adipogenesis in MSCs. The presence of myostatin downregulated the expression of adipocyte markers, induced SMAD3 phosphorylation, and activated β-catenin in human BMSCs [49]. As previously mentioned, BMP7 triggered the adipocyte commitment of C3H10T1/2  cells. Myostatin prevented BMP7 signaling by blocking BMP7 receptor binding, resulting in reducing adipogenesis in C3H10T1/2 cells [38]. Myostatin is an important factor in brown adipocyte lineage determination. *Mstn*^−/−^ mouse embryonic fibroblasts (MEFs) had enhanced expression levels of brown adipocyte markers including UCP1, PRDM16, and PGC1 compared with the control group undergoing adipogenic differentiation [72]. From the above statement, we can conclude that myostatin plays an extremely complicated role in the adipocyte commitment of MSCs .

## Conclusion

TGF-β superfamily signaling has an important function in regulating the adipocyte commitment of MSCs. TGF-βs ligands such as TGF-β and myostatin use SMAD2/3 signaling to regulate adipocyte commitment. However, BMPs ligands such as BMP2/4/7 use SMAD1/5/8 signaling to regulate adipocyte commitment. BMP4 signaling is explicitly sufficient to induce adipocyte lineage determination and the formation of brown adipocytes in MSCs. BMP2 and myostatin signaling have an effect on the adipocyte commitment of MSCs. Some studies found that BMP2 or myostatin signaling can promote adipocyte commitment in MSCs, while animal experiments showed the same results. However, some studies found that BMP2 or myostatin signaling can inhibit this commitment. It is unclear whether they promote adipocyte commitment or inhibit it. It is well known that BMP2 and myostatin signaling were originally identified based on their ability to induce osteogenesis and myogenesis inhibition. BMP2 and myostatin signaling may play a small part in adipocyte commitment, perhaps due to different donor lines, the concentrations of an adipogenic induction cocktail, or serum types in culture medium. Previous articles have demonstrated that TGF-β signaling promoted the proliferation of MSCs and suppressed the adipocyte commitment of MSCs by inhibiting C/EBPs and PPARγ expression. Recent studies have showed that TGF-β promoted adipocyte commitment. The difference was that they studied the adipocyte commitment of BMSCs isolated from different species: mice and humans [45, 47]. MSC origin may affect adipocyte commitment under TGF-β signaling. Various clones isolated from human BMSC lines have different differentiation capacities, which may be the factor affecting the commitment event. More studies are required to address the effect of BMP2, TGF-β, and myostatin on adipocyte commitment in MSCs. BMP7 mainly promotes the commitment of MSCs to a brown adipocyte lineage. Other TGF-β superfamily members such as BMP3, BMP6, and GDF11 have been evaluated as to whether they affect commitment. BMP3 stimulated the proliferation rather than the commitment of MSCs through TGF-β/activin signaling [73]. BMP6 is highly homologous with BMP7, but BMP6 mainly affects the adipocyte differentiation process of MSCs rather than the commitment process. BMP6 governs the lineage adipocyte commitment of other cell types. BMP6 stimulation in muscle precursor cells C2C12 was sufficient to induce brown fat determination [74]. Little research has been performed on GDF11, but a recent article discussed the effect of GDF11 on adipocyte commitment. The data indicated that GDF11 inhibited adipogenic differentiation in human MSCs by activating SMAD2/3-dependent TGF-β signaling [75]. It is becoming clear that R-SMADs have an impact on the commitment of MSCs, but we still know very little about Co-SMAD (SMAD4). SMAD4 is common to all receptor pathways, and its mechanism is complicated. A recent article reported that SMAD4 depletion in C3H10T1/2 cells and hASCs reduced the nuclear retention of TAZ, a transcriptional coactivator with a PDZ-binding motif, resulting in enhanced adipogenesis of the MSCs through dissociating TAZ–PPARγ interactions [76]. However, this study runs counter to the above conclusion [30]. Both studies used RNA interference to silence *SMAD4* in C3H10T1/2 cells. One study added BMP2 protein but the other did not, causing a different outcome because the function of SMAD4 was independent of the TGF-β signaling in some cases, and sometimes BMP2 had a role in adipocyte commitment independent of TGF-β/SMAD signaling. PPARγ is a master transcription factor in adipocyte commitment. BMP2/4 signaling induces adipocyte commitment of MSCs by activating PPARγ expression. A recent novel study reported the epigenetics of adipogenic commitment under TGF-β/SMAD signaling. *miR-20a* promoted C3H10T1/2 cells to commit, and this function may depend upon its direct inhibitory effect on lysine-specific demethylase 6B (KDM6B) and TGF-β signaling [77]. Thus far, the research on adipocyte commitment in MSCs has had some limitations: (1) the disunified source and unclear features of MSCs, (2) the limited research available on the role of the entire TGF-β superfamily, and (3) the unclear relationships between the TGF-β superfamily members. Perhaps future studies should focus more on these limitations.

The TGF-β superfamily members have an effect on adipocytokines secreted from WAT and BAT as well as beige adipose tissue, influencing white-to-brown transition and disease. Future studies of TGF-β superfamily signaling will offer new approaches in treating obesity, diabetes mellitus, and obesity-related metabolism syndrome.

## Data Availability

Not applicable.

## Abbreviations

AP2: Adipocyte protein 2
BAT: Brown adipose tissue
BMPs: Bone morphogenetic proteins
BMSCs: Bone marrow stromal stem cells
C/EBPα: CCAAT/enhancer binding protein-α
CS: Calf serum
FBS: Fetal bovine serum
GDFs: Growth differentiation factors
hASCs: Human adipose stem cells
KDM6B: Lysine-specific demethylase 6B
LOX: Lysyl oxidase
MEFs: Mouse embryonic fibroblasts
MSCs: Mesenchymal stem cells
PPARγ: Peroxisome proliferator-activated receptor-γ
SVF: Stromal vascular fraction
TCF21: Transcription factor 21
TGF-β: Transforming growth factor-β
TPT1: Translationally controlled tumor protein 1
UCP1: Uncoupling protein 1
WAT: White adipose tissue
